# Treatment with ensartinib improves postoperative survival in a patient with stage IIB LUAD carrying a rare ALK mutation: case report

**DOI:** 10.1515/biol-2025-1266

**Published:** 2026-03-13

**Authors:** Qiang Guo, Xiang-Yu Luo, Hua Liu, Min Zeng, Jun Zhou, Jun Zhang, Jia-Long Guo, Cheng-Yi Lin

**Affiliations:** Department of Cardiothoracic Surgery, Taihe Hospital, Hubei University of Medicine, Shiyan, China

**Keywords:** ensartinib, LUAD, TOGARAM2-ALK, lymph node metastasis, micropapillary

## Abstract

A patient with stage IIB LUAD carrying a rare TOGARAM2-ALK mutation received long-term postoperative oral ensartinib therapy and showed no significant systemic progression or recurrence. A chest CT scan performed 3 days before admission revealed a 2.5 × 2.1 cm space-occupying lesion in the posterior segment of the right upper lobe, and thoracoscopic right upper lobectomy with mediastinal lymph node dissection was completed on the fourth day after admission. Postoperative pathology confirmed infiltrating adenocarcinoma in the right upper lobe of the lung, with predominant acinar growth, and additional papillary, micropapillary, and adherent components. The tumor measured 2.2 cm in diameter with evidence of airway spread, and right paratracheal lymph nodes showed metastatic involvement. Genetic testing detected a TOGARAM2-ALK (ch2:29244065) fusion mutation. The patient recovered well and was discharged after four postoperative days. During more than four years of follow-up, long-term ensartinib therapy was associated with stable disease without recurrence, offering potential clinical insights for managing LUAD with rare *ALK* variants.

## 1 Background

Lung cancer remains one of the most prevalent malignant tumors worldwide, with mortality rates consistently ranking highest among all cancers [1], [2], [3]. Lung adenocarcinoma (LUAD), a predominant subtype, has benefited from significant advancements in treatment [1], [2], [3], [4], evolving from platinum-based chemotherapy to targeted therapies, such as Epidermal Growth Factor Receptor (EGFR) inhibitors (such as gefitinib), and more recently to immunotherapies directed at Programmed Cell Death Protein 1 (PD-1)/Programmed Death-Ligand 1 (PD-L1) checkpoints. These outcomes have substantially improved outcomes in advanced disease.

This case report presents a rare case of intermediate-to-advanced LUAD harboring a TOGARAM2-ALK fusion, involving the Tetratricopeptide Repeat-Containing Gamma-Tubulin Ring Complex Associated Protein 2 (*TOGARAM2*) and Anaplastic Lymphoma Kinase (*ALK*). Postoperative pathological examination revealed infiltrating adenocarcinoma of the upper lobe of the right lung, with a predominantly acinar pattern, and additional components of papillary, micropapillary, and adherent components. The tumor measured 2.2 cm in diameter, with intra-airway spread but no pleural or definitive nerve invasion. The bronchial resection margin was negative. Metastasis was identified in the right paratracheal lymph node, while all other nodes were uninvolved. Genetic testing confirmed a rare TOGARAM2-ALK mutation (ch2:29244065). Following long-term ensartinib therapy administered outside the hospital, the patient has shown no significant disease progression or recurrence over a follow-up period exceeding four years, demonstrating a considerably improved prognosis for this uncommon mutation subtype. This case underscores the importance of molecular profiling in guiding targeted therapy, and highlights the potential for prolonged disease control in patients with rare genetic alterations.

## 2 Case description

**Figure 1:**
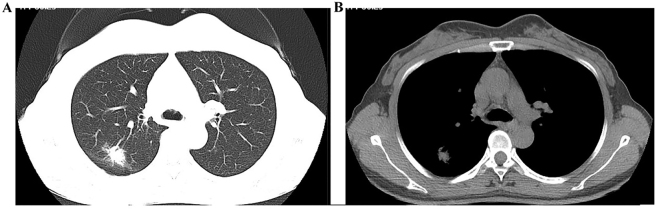
Chest CT scan demonstrating a right upper-lobe pulmonary nodule. (A) Lung window; (B) mediastinal window.

A 47-year-old female patient underwent a chest computed tomographic (CT) scan on August 25, 2021, which revealed a 2.5 cm × 2.1 cm space-occupying lesion in the posterior segment of the right upper lobe, adjacent to the pleura, suggestive of peripheral lung cancer (Figure 1A and B). The patient was admitted on August 28, 2021. The patient denied cough, sputum, chest tightness, chest pain, fever, night sweats, hoarseness, dyspnea, or other symptoms. She has no history of hypertension, diabetes, hepatitis, tuberculosis, or drug allergy. Physical examination showed T 36.6 °C, P 60/min, R 20/min, BP 109/68 mmHg. No jaundice, petechiae, or superficial lymphadenopathy were present. Lung auscultation revealed coarse breath sounds without rales. There was no precordial bulge and no palpable thrill. The heart rate was normal, with no pathological murmurs. The abdomen was flat and soft, without tenderness or rebound pain; the liver, spleen, and costal margins were not palpable, and there was no percussion tenderness over either renal angle. No lower-limb edema was noted.

Further evaluations were completed after admission. Abdominal ultrasound indicated a solid nodule in the right liver lobe consistent with hemangioma, and polypoid lesions in the gallbladder. The spleen, pancreas, and portal vein were unremarkable. Echocardiography demonstrated mild mitral and tricuspid regurgitation. The brain CT scan was unremarkable. Based on these findings, the clinical stage was determined to be cT1cNxM0. Thoracoscopic right upper lobectomy with mediastinal lymph node dissection was performed on September 1, 2021.

**Figure 2:**
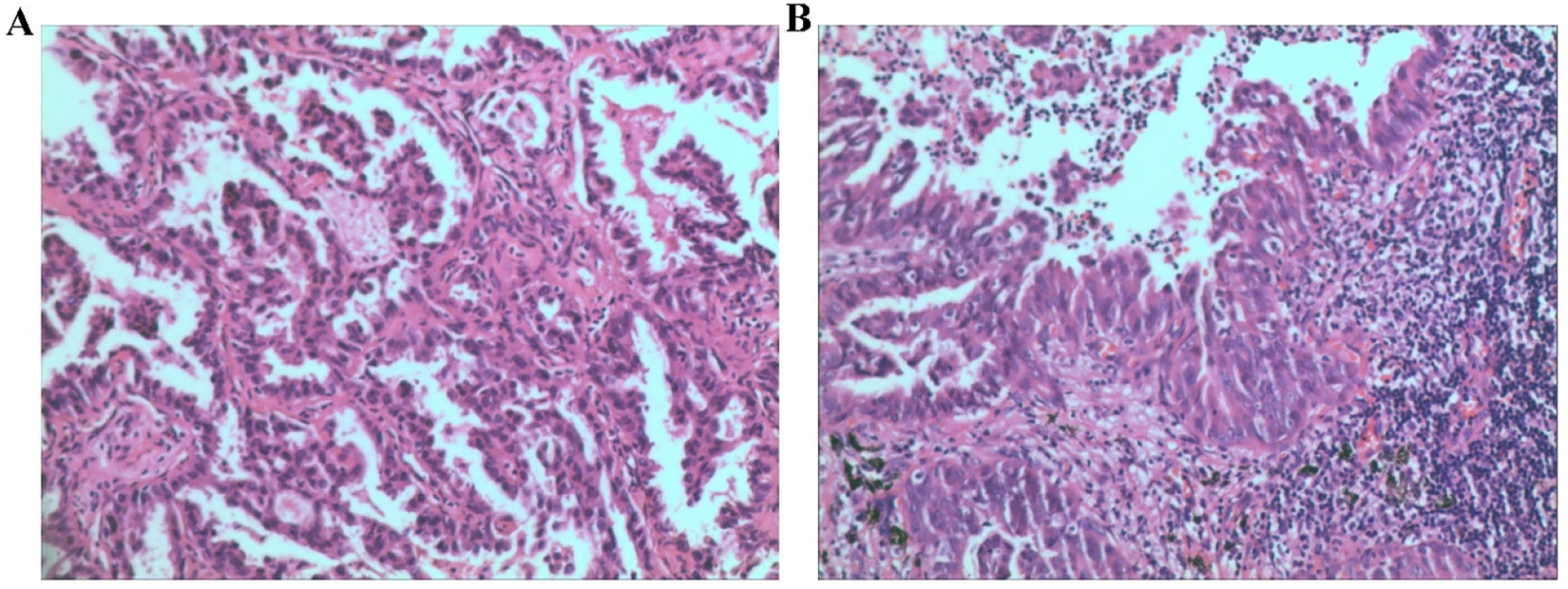
Pathological findings. (A) Intraoperative frozen-section pathology; (B) postoperative histopathology.

After induction of general anesthesia and endotracheal intubation, the patient was placed in the left lateral decubitus position and routinely disinfected. Thoracoscopic access was established using an incision at the fourth intercostal space along the right anterior axillary line and a 2-cm port at the seventh intercostal space. No pleural effusion or adhesions were observed. The lesion was located in the posterior segment of the right upper lobe, where a palpable 2.5-cm nodule with no apparent surface collapse was identified. The lesion and adjacent lung tissue were elevated, and the lung tissue at the base was resected using a 45-mm endoscopic linear stapler. Intraoperative frozen section confirmed invasive adenocarcinoma (Figure 2A). A thoracoscopic right upper lobectomy with mediastinal lymph node dissection was then completed. The interlobar fissure, superior pulmonary vein, and the right superior lobar artery with its branches were divided using a 45-mm linear stapler. The right superior lobar bronchus was isolated and transected with the same device. After re-expansion of the middle and lower lobes, the right upper lobe was removed. Superior and inferior paratracheal, subcarinal, and hilar lymph nodes were routinely dissected. A chest tube was placed, and the thorax was closed in the standard manner.

**Figure 3:**
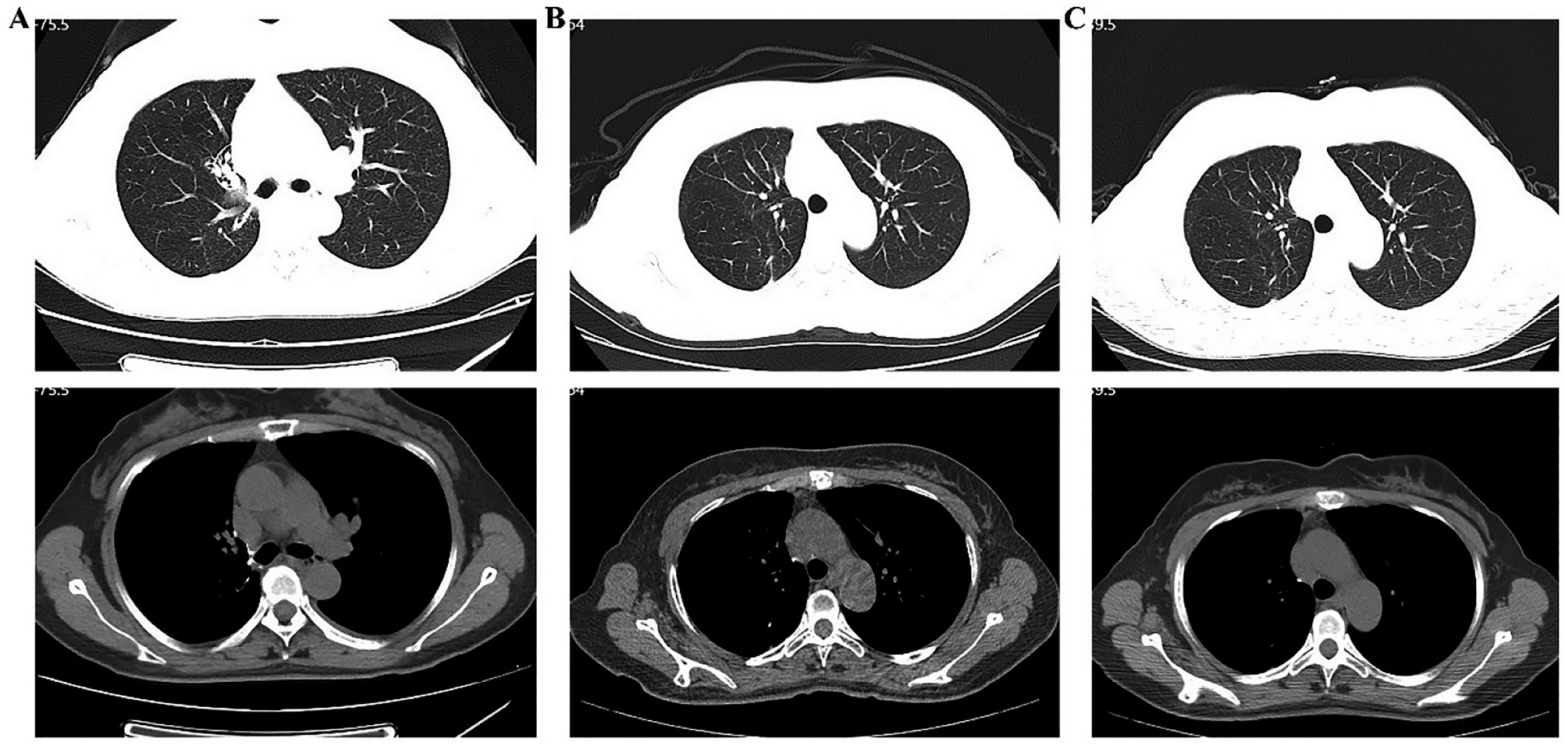
Chest CT scans showing postoperative changes in the right upper lung. (A) July 30, 2024; (B) January 6, 2025; (C) July 24, 2025.

**Figure 4:**
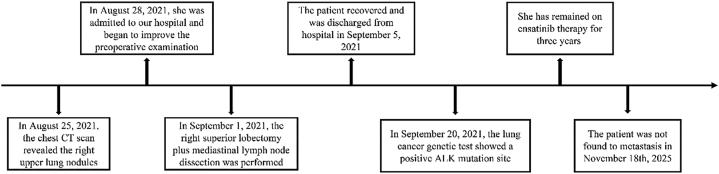
Timeline of the patient’s diagnostic evaluation and treatment from August 25, 2021, to November 18, 2025.

On postoperative day 3, the patient’s pathological stage was determined as pT1cN1M0, stage IIB. The resected specimen showed infiltrating adenocarcinoma of the right upper lobe (Figure 2B), predominantly acinar (approximately 70 %), with papillary, micropapillary, and adherent components. The tumor measured 2.2 cm with intra-airway spread; no vascular invasion, pleural involvement, nerve invasion, or bronchial margin infiltration was identified. Metastasis was found in one right paratracheal lymph node (1/1), while all other lymph nodes were negative. The patient recovered well and was discharged on postoperative day 4 on September 5, 2021. Genetic testing performed by BGI Genomics Co., Ltd. (Shenzhen, Guangdong Province, China) using high-throughput sequencing confirmed aTOGARAM2-ALK fusion mutation on September 20, 2021. Sequencing covered single-nucleotide variants (SNVs), insertion–deletion variants (InDels), fusions, and copy number variations. The mutation was located at ch2:29244065 with a variant allele frequency of 8.44 %. After discussion with the patient and her family, ensaratinib (225 mg/day) was initiated on September 30, 2021. During treatment, regular follow-up calls were conducted, and chest CT scans were obtained every six months. No gastrointestinal symptoms, rash, fatigue, or pneumonia were reported between September 30, 2021, and September 30, 2024. CT scans on July 30, 2024, January 6, 2025, and July 24, 2025, demonstrated only postoperative changes in the right upper lobe (Figure 3A–C). Throughout follow-up, no significant tumor progression or recurrence was observed, as shown in the timeline (Figure 4).

**Informed consent:** Informed consent has been obtained from all individuals included in this study.

**Ethical approval:** The research related to human use has been complied with all the relevant national regulations, institutional policies and in accordance with the tenets of the Helsinki Declaration, and has been approved by the Ethics Committee of Taihe Hospital.

## 3 Discussion

Driver gene testing in LUAD is essential for guiding precision targeted therapy. Several oncogenic alterations, including those of *EGFR*, *ALK*, c-ros oncogene 1 receptor tyrosine kinase (*ROS1*), and Rearranged during Transfection (*RET*), are well-established. *ALK* mutations account for approximately 3–7 % of mutations in non-small cell lung cancer, and occur more frequently in non-smokers or light smokers, younger patients, and those with adenocarcinoma. ALK-rearranged LUAD represents a heterogeneous molecular subgroup driven by fusions involving the ALK receptor tyrosine kinase, located on chromosome 2. Historically, those ALK-positive and treated with cisplatin demonstrated poor outcomes; however, the introduction of ALK inhibitors has markedly improved survival. Multiple generations of ALK inhibitors are now available, including first-generation crizotinib, second-generation ensartinib, third-generation lauratinib, and fourth-generation agents such as loptinib. Known *ALK* fusion partners include *TACR1, DYSF, KLC1, EML4*, and *HIP1*, with *EML4* being the most common.

The TOGARAM2-ALK fusion is an exceedingly rare molecular event. The *TOGARAM2* gene, located on chromosome 2, plays a role in microtubule cytoskeletal organization. A PubMed search revealed no prior reports of postoperative patients with LUAD and TOGARAM2-ALK fusion treated with ensaratinib, and follow-up outcomes in patients with ALK positivity are limited. Only one previously published case by Yan et al. involved a 27-year-old female patient with advanced LUAD harboring TOGARAM2-ALK fusion and brain metastases [5]. After oral treatment with loratinib, the patient’s brain lesions remained stable without progression. In our case, chest CT revealed a 2.5 × 2.1 cm posterior right upper-lobe lesion suspicious for malignancy. Surgery was performed on day 4 of admission. Pathology confirmed invasive adenocarcinoma with papillary, micropapillary, and adherent components, measuring 2.2 cm in diameter with airway spread and right paratracheal lymph-node metastasis. Genetic testing identified a TOGARAM2-ALK fusion consistent with mutational characteristics previously described [6]. The patient received long-term ensartinib therapy and demonstrated no significant progress or recurrence during a 4-year follow-up period, highlighting the potential efficacy of targeted therapy in this rare fusion subtype.

Current evidence demonstrates that ALK inhibitors provide substantial clinical benefit in ALK-positive LUAD [5], [6], [7], [8], [9], [10], [11], [12], [13], [14]. However, no published reports describe postoperative ensartinib therapy. This report describes a patient with a surgically confirmed stage IIB LUAD and a novel ALK rearrangement variant, TOGARAM2-ALK, who achieved sustained disease control with ensartinib. Although this second-generation ALK inhibitor can significantly prolong survival, it may cause hepatic toxicity, gastrointestinal symptoms, rash, fatigue, and pneumonia. When such adverse effects occur, appropriate management strategies are essential to optimize treatment benefits and minimize toxicity. In this case, the patient received long-term ensartinib therapy outside the hospital, and showed no progression or recurrence over a 4-year follow-up period. This case report provides clinicians with experience-based insight into the management of ALK-positive LUAD with rare fusion variants.
